# Quality Indicators for Transition from Pediatric to Adult Care for Youth With Chronic Conditions: Proposal for an Online Modified Delphi Study

**DOI:** 10.2196/60860

**Published:** 2024-09-10

**Authors:** Alene Toulany, Dmitry Khodyakov, Sarah Mooney, Lisa Stromquist, Katherine Bailey, Claire EH Barber, Michelle Batthish, Kristin Cleverley, Gina Dimitropoulos, Jan Willem Gorter, Danijela Grahovac, Ruth Grimes, Beverly Guttman, Michèle L Hébert, Tomisin John, Lisha Lo, Dorothy Luong, Laura MacGregor, Geetha Mukerji, Jacklynn Pidduck, Vjura Senthilnathan, Rayzel Shulman, Patricia Trbovich, Sarah EP Munce

**Affiliations:** 1 Temerty Faculty of Medicine University of Toronto Toronto, ON Canada; 2 Department of Adolescent Medicine The Hospital for Sick Children Toronto, ON Canada; 3 Institute of Health Policy, Management and Evaluation University of Toronto Toronto, ON Canada; 4 RAND Santa Monica, CA United States; 5 Stollery Children’s Hospital Alberta Health Services Edmonton, AB Canada; 6 Alberta Strategy for Patient Oriented Research Support Unit University of Calgary Calgary, AB Canada; 7 Children’s Healthcare Canada Ottawa, ON Canada; 8 Department of Medicine University of Calgary Calgary, AB Canada; 9 Department of Pediatrics McMaster University Hamilton, ON Canada; 10 Centre for Addiction and Mental Health Toronto, ON Canada; 11 Lawrence Bloomberg Faculty of Nursing University of Toronto Toronto, ON Canada; 12 Department of Rehabilitation, Physical Therapy Science & Sports Wilhelmina Children's Hospital Utrecht Netherlands; 13 CanChild Centre for Childhood Disability Research McMaster University Hamilton, ON Canada; 14 Children’s Healthcare Canada National Health Hub in Transition Hamilton, ON Canada; 15 Canadian Pediatric Society Winnipeg, MB Canada; 16 Provincial Council for Maternal and Child Health Toronto, ON Canada; 17 Faculty of Rehabilitation Medicine Department of Occupational Therapy University of Alberta Edmonton, AB Canada; 18 Division of Adolescent Medicine The Hospital for Sick Children Toronto, ON Canada; 19 Centre for Quality Improvement and Patient Safety University of Toronto Toronto, ON Canada; 20 Rehabilitation Sciences Institute University of Toronto Toronto, ON Canada; 21 Martin Luther University College Waterloo, ON Canada; 22 Women’s Institute of Health Systems Solutions and Virtual Care Women’s College Hospital Toronto, ON Canada; 23 IWK Health Halifax, NS Canada; 24 Division of Endocrinology Department of Paediatrics University of Toronto Toronto, ON Canada; 25 Patient Safety and Quality Improvement North York General Hospital Toronto, ON Canada; 26 KITE Toronto Rehabilitation Institute University Health Network Toronto, ON Canada; 27 Department of Occupational Science and Occupational Therapy University of Toronto Toronto, ON Canada

**Keywords:** transition to adult care, adolescent health, chronic conditions, quality indicators, consensus, caregivers, adolescent, stakeholder engagement, patient engagement, Delphi

## Abstract

**Background:**

The transition from pediatric to adult care poses a significant health system–level challenge impeding the delivery of quality health services for youth with chronic health conditions. In Canada and globally, the transition to adult care is regarded as a top priority in adolescent health in need of readily applicable, adaptable, and relevant national metrics to evaluate and benchmark transition success across disease populations and clinical care settings. Unfortunately, existing literature fails to account for the lack of engagement from youth and caregivers in developing indicators, and its applicability across chronic conditions, primary care involvement, and health equity considerations.

**Objective:**

Our proposed study aims to establish a consensus-driven set of quality indicators for the transition to adult care that are universally applicable across physical, developmental, and mental health conditions, clinical care settings, and health jurisdictions.

**Methods:**

Using an integrated knowledge translation (iKT) approach, a panel comprising youth, caregivers, interdisciplinary health care providers, and health system leaders will be established to collaborate with our research team to ensure that the study methodology, materials, and knowledge dissemination are suitable and reflect the perspectives of youth and their families. We will then conduct an iterative 3-round Online Modified Delphi (OMD) study (n=160) to (1) compare and contrast the perspectives of youth, caregivers, health care providers, and health system leaders on quality indicators for transition; and (2) prioritize a key set of quality indicators for transition applicable across disease populations that are the most important, useful, and feasible in the Canadian context. Using the RAND/UCLA Appropriateness Method (RAM) multistage analytic approach, data from each panel and stakeholder group will be examined separately and compared to establish a key set of indicators endorsed by both panels.

**Results:**

The study is funded by the Canadian Institutes of Health Research and Physicians Services Incorporated.

**Conclusions:**

This study will produce quality indicators to evaluate and inform action equitably to improve transition from pediatric to adult care for youth and their families in Canada.

**International Registered Report Identifier (IRRID):**

PRR1-10.2196/60860

## Introduction

Transition from pediatric to adult care services is a challenging and complex process for many youths with chronic physical, developmental, and mental illnesses, and their families [1-3]. Up to 15% of youth in North America are affected by a chronic condition that will require transition to adult care, typically at the age of 18 in Canada [4]. Over 98% of these youth are expected to reach age 20 years and transition to adult health care services [5]. In Canada alone, around 70,000 of these transfers occur every year [6]. These numbers are growing due to advancements in health care which have led to improved survival into adulthood [2]. Chronic conditions make up about 58% of annual total direct health care costs in Canada [7]. In 2016, US $179.6 million was spent on direct health care costs for youth with cirrhosis alone [7]. Additionally, many youths with chronic conditions exhibit poor health outcomes following their transition to the adult health care system (eg, an increase in emergency department visits, hospitalizations, missed appointments, and relapses in disease states) and community services [8-13]. For example, youth living with childhood chronic and complex physical disabilities, specifically cerebral palsy, spina bifida, and acquired brain injuries are expected to be hospitalized once every 6.8 years after transfer, which is 9 times higher than the general population [14].

Significant barriers to successful transition include inconvenient timing during a critical period in adolescent physical and psychosocial development, concurrent life milestones, poor youth and caregiver readiness, inadequate care coordination, underlying differences in pediatric versus adult approaches to care, lack of provider comfort and expertise in managing complex child-onset conditions, limited time and resources for transition navigation, and variation in governance structures and funding models [15]. Accordingly, transition to adult care has been recognized as a priority area in adolescent health care by the Canadian Pediatric Society [16] and highlighted as 1 of 5 domains of need and priority in adolescent health investment in Canada [9]. Significant gaps exist in evaluating and measuring health system performance (ie, the extent to which a health system achieves its goals to improve quality, efficiency, and accessibility) in transition [16]. One major barrier is the absence of universal measures to assess structures, processes, and outcomes in transition care. In practice, there are major inconsistencies in how measurement of transition is approached as there are currently no national Canadian standards informing measurement-based care in transition. A critical step in improving the quality of care delivered for youth with chronic conditions and their families is to develop a systematic approach to measurement in transition through a key set of quality indicators [17]. Quality indicators are defined as robust measures used to evaluate health care delivery, processes, and outcomes to enhance quality of care [17,18] and benchmark performance [19,20], informed by key knowledge users. When applied and interpreted carefully, quality indicators could reveal inequities in access, delivery, and outcomes of transition services while highlighting national health system–level practice changes needed to address gaps.

Results of a recent systematic review conducted by our research team identified 169 quality indicators for transition to adult care for youth with diverse chronic conditions; however, significant gaps were found in the process to transition to adult care [21-23]. Notably, the lack of quality indicators focused on equity during the transition period demonstrates that this is an aspect that is neglected in both transition research and the provision of care. Additionally, many studies were limited to physical illnesses (eg, inflammatory bowel disease, sickle cell disease) and excluded mental illnesses and developmental disabilities [24-26]. Consequently, there are no robust quality indicators which can be used in transition across all chronic conditions, hindering our ability to broadly evaluate transition to adult care at a health system level in Canada. While the majority of quality indicators identified in the review were patient-centered metrics, their development rarely involved youth and caregivers [24,25,27]. When included, youth and caregivers were often outnumbered by clinicians, making them less likely to raise their concerns [28]. Previous studies have suggested the priorities of youth with chronic illnesses are different from those of their health care team [29]. Several reviews have concluded that youth want to be involved not only in their own transition planning [30,31] but also in designing and participating in transition research [32]. Engaging youth and caregivers in the design and execution of transition research can increase its relevance for other patients receiving transition services [33]. To prevent the development of poorly actionable quality improvement initiatives and ensure they are meaningful to patients, transition research must be grounded in the unique perspectives and experiences of youth and their caregivers. Building on our previous work [21], this proposed study addresses significant gaps in the literature, particularly the lack of meaningful youth and caregiver engagement in indicator development, applicability across chronic health conditions, community primary care involvement, and health equity considerations.

This study aims to establish a key set of consensus-derived quality indicators for transition to adult care that are applicable across chronic physical, developmental, and mental health conditions, clinical care settings, and health jurisdictions. Following the establishment of an integrated knowledge translation panel, we aim to use an iterative online-modified Delphi study design to (1) compare and contrast the perspectives of youth, caregivers, health care providers, and health system leaders or decision makers on quality indicators for transition; and (2) prioritize a key set of quality indicators for transition applicable across disease populations and care settings in the Canadian context.

## Methods

### Study Design

Using an integrated knowledge translation (iKT) approach [34], an iKT panel comprising youth, caregivers, interdisciplinary health care providers, and health system leaders will be established at the outset of the project. The iKT panel will collaborate with the research team to ensure that the study methodology and materials are appropriate and youth- and family-centered. We will then conduct a 3-round Online Modified Delphi (OMD) study to prioritize a set of quality indicators for transition across chronic physical, developmental, and mental health conditions that are most important, useful, and feasible for Canadians. Findings will be reported in accordance with the ACcurate COnsensus Reporting Document (ACCORD) reporting guideline [35].

### iKT Panel

iKT is defined as a dynamic collaboration between researchers and knowledge users, or those with the authority to enact change, in the synthesis, dissemination, and application of knowledge. An iKT panel with diverse representation will be established and comprise an equal distribution of youth, caregivers, health care providers, and health system leaders in addition to the research team. Panel members will be recruited using the research team’s pre-existing relationships with key knowledge users, including leading organizations in child and youth health in Canada. Purposive recruitment will ensure knowledge users with varied backgrounds who can represent transitional care issues that are specific to rural, remote, Francophone, Indigenous, and immigrant communities are included. We will use the best practice activities outlined in the Canadian Institutes of Health Research’s (CIHR’s) Strategy for Patient-Engagement in Research, in addition to the Ontario Brain Institute (OBI) framework to facilitate engagement with participants and to collaborate [36-38].

The iKT panel will meet on a quarterly basis and be actively engaged throughout the research process. Decision-making processes will occur through active discussion in panel meetings and written feedback. The primary activity of the iKT panel will be to work with the research team to refine the previously identified quality indicators for transition [21] (n=169) to present to the OMD participants minimizing participant burden. Further, the iKT panel will be responsible for finalizing the criteria (eg, importance, usefulness, and feasibility) used to rate quality indicator categories and determining the decision rules for achieving consensus. The panel will also help to inform participant recruitment strategies, consult on the development of supplemental materials for participants in the OMD study (eg, project goals, processes, and timeline), ensure concepts and materials used in the OMD are easily understood and methods used are appropriate for youth and caregivers, and inform the strategies for knowledge dissemination.

### OMD Study

Once the quality indicators for transition have been refined, the rating criteria have been finalized, and the decision rules for achieving consensus have been established, we will conduct a 3-round OMD study to prioritize the quality indicators. Each category of quality indicator will be individually rated on the proposed categories of importance and feasibility. These rating scales have been adapted from a previous study aimed at developing quality indicators to evaluate health system performance [39]. The OMD process will follow the RAND guidance for conducting Delphi studies and consists of 2 concurrently run panels [40].

The OMD panels will be administered using *ExpertLens,* an online platform for conducting OMD studies developed by RAND, which has been instrumental in advancing consensus-building methodologies. *ExpertLens* has been used previously for quality indicator development in Canada [39,41] and allows for conducting RAND/UCLA Appropriateness panels (a gold standard for developing clinical guidelines and quality indicators) completely online [42]. The RAND/PPMD Patient-Centeredness Method (RPM)—a recently developed approach for engaging patients and caregivers in guideline development—also used the *ExpertLens* platform, which shows the feasibility and benefits of soliciting patient and caregiver input in clinical processes [43,44].

#### OMD Panels and Participants

We will assemble 2 panels that will be administered concurrently using identical data collection protocols. To maintain balanced panels in all rounds, the 2 panels will be aimed to include equal representations from each expert group [40]. A 2-panel approach ensures methodological robustness and strengthens confidence in our results [40]. Our goal is to recruit approximately 160 participants across the 4 knowledge user groups or “types of experts” (approximately 40 participants per type):

1. Youth (18-24 years old) with any chronic physical, developmental, or mental health condition who are actively navigating or have recently completed their transition to adult care.
2. Caregivers of youth who are currently transitioning or have transitioned to adult care in the last 6 years.
3. Multidisciplinary health care providers from across the continuum of transition, including pediatric physicians, adult physicians, primary care providers, nurses, social workers, mental health professionals, and transition coordinators.
4. Health system leaders such as individuals in child health management or administrative roles, policy makers, decision makers, and community health agency leaders.

Each panel will include 80 participants to increase panel representativeness, engage experts in meaningful online discussions, and explore differences between and within expert groups [45]. Because attrition is common in Delphi panels, we anticipate that about 50-60 participants will complete all OMD rounds in each panel [40].

Similar to recruitment for the iKT panel, we will leverage pre-existing relationships with the Children's Healthcare Canada’s (CHC) Health Hub in Transition, the Canadian Paediatric Society (CPS), the Provincial Council for Maternal and Child Health (PCMCH), and other national networks. To minimize the risk of selection bias, we will expand recruitment to community organizations and social media platforms to reach youth and caregivers who may have had an unsuccessful transition or are not connected with a health care team. We may expand our recruitment strategies after consultation with the iKT panel.

Interested participants will be asked to complete a registration survey, provide basic information about them, and suggest quality indicators the panelists should consider. The main selection criteria for OMD participants will include experience with transition care in their respective specialty, navigating transition services, and geographic, practice setting, biological (eg, sex and age), and sociodemographic (eg, gender, race, and ethnicity) diversity. A purposeful recruitment of diverse participant demographics will ensure that an appropriate key set of quality indicators will be developed with equity considerations. Nationally relevant benchmarks require representation from diverse geographical areas (ie, each province and territory, both rural and urban settings), practice settings, and health care organizations [43]. We will use an equity lens to amplify the voices of youth and caregivers that are marginalized, including Black, Indigenous, and LGBTQQIP2SAA (lesbian, gay, bisexual, transgender, queer, questioning, intersex, pansexual, two-spirited, and asexual) individuals, who may have unique experiences with transition. We will aim for at least 25% of participants self-identifying as individuals that are marginalized.

We will review the list of potential participants and identify those who best meet our selection criteria. Selected potential participants will be invited to participate and those who agree will participate in the 3-round OMD. Selected experts from each expert group will be randomly assigned to 1 of the 2 panels, with a goal of achieving balanced panels [46,47]. We will supply participants with preparatory materials that explain the study aims, provide descriptions of candidate quality indicators, and list the rating criteria.

#### 3-Round OMD

Participants in both panels will complete a 3-round OMD process where they will be rating and discussing the candidate quality indicator.

In round 1, expert panelists will be presented with a brief description of each candidate quality indicator category and asked to rate the category on a questionnaire on 2 criteria:

1. Importance: How important is this category of quality indicators in evaluating the transition from pediatric to adult care?
2. Feasibility: How easy is it to obtain this category of quality indicators when evaluating the transition from pediatric to adult care?

Participants will use a 9-point Likert scale (eg, 1=not very important; 9=very important) to rate candidate quality indicators and explain their numeric responses by stating what factors affected their ratings. Participants will be asked to provide recommendations regarding any additions or deletions to the list of candidate quality indicators.

In round 2, participants will see a bar chart for each question demonstrating their responses from round 1 in relation to panel responses. A statement will be displayed for each chart indicating whether agreement between the panel and each of the 4 expert groups was achieved and whether participants considered each quality indicator category to be important and feasible. Further, a comment summary of the major themes identified will be provided for low (1-3), uncertain (4-5), and high (7-9) ratings.

Participants will then engage in an online discussion about round 1 results through an asynchronous discussion board. The online discussion boards will be moderated by members of the research team to ensure they remain constructive. A moderator guide developed specifically for *ExpertLens* discussions will be followed [44]. Round 2 discussion data will be used to provide a more nuanced description of knowledge user perspectives and the reasons why expert users agree or disagree with each other.

In round 3, using a questionnaire, participants will have the option to update their round 1 responses according to the feedback and discussions obtained during round 2. Round 3 rating data will be used to determine if there is consensus among diverse experts and prioritize proposed indicators.

Each round will take place over 7-14 days to ensure panelists have ample time to participate and periodic reminders will be sent to maximize participation. The overall time commitment for all 3 rounds will be 3-4 hours in total, with Rounds 1 and 3 taking approximately 1 hour each and round 2 approximately 2 hours. Those completing all 3 rounds will receive compensation for their participation.

### Analysis

We will use a previously developed multistage analytic approach to analyzing *ExpertLens* data [28]. We will examine data from each panel and expert group separately and then compare the results to establish a prioritized set of proposed quality indicators using a 3-step approach described below.

First, we will use descriptive statistics (ie, medians and interquartile ranges) to identify how participants within each panel and across the 2 panels viewed each quality indicator category in rounds 1 and 3.

Second, we will use the RAND/UCLA Appropriateness Method (RAM) manual’s [47] approach to determine if consensus was reached within each expert group and panel after rounds 1 and 3. *ExpertLens* will automatically do this and display round 1 results to participants in rounds 2 and 3. Round 3 ratings will be used to determine the final study results and address aims. RAM [47] uses the distribution of panel responses to determine if participants agree or disagree with each other and relies on the median value to indicate if the panel’s determination is positive, negative, or uncertain.

Third, we will use round 3 data for each indicator category to determine which category is endorsed by each panel. We will identify indicator categories rated highly (median score of 6.5 and above without disagreement) on all rating criteria and automatically include them for further consideration [42].

Finally, we will determine the replicability of the final panel results by calculating a κ coefficient for each indicator category. If the κ statistic is at least moderate (0.41-0.60), we will consider the level of replicability of panel ratings to be acceptable [48]. This threshold is considered conservative. Conducting 2 concurrently run panels will not only help us identify indicators with the highest level of agreement but also increase confidence in our final study results. The final list of endorsed indicator categories will include those rated highly on importance and feasibility by both panels.

To better explain panel and expert group ratings of different indicators, we will analyze participants’ explanations of ratings and discussion comments via content analysis [44,47,49]. For each panel separately, we will group all comments for each indicator and rating criterion based on the numeric ratings to which they refer; we will also group all discussion comments by the indicator on which they focus. The coding team will review and code all qualitative comments inductively to identify themes that could be used to explain why a certain quality indicator was considered important, useful, or feasible or if experts have similar perspectives [50]. All coding results will be reviewed by DK to ensure consistency in applying the codebook; AT and other study team members will review coding results to ensure the correct clinical interpretation of comments and appropriateness of conclusions.

### Patient and Public Involvement

This study will be heavily influenced by patient and public involvement. Key knowledge users in transition to adult care—youth, caregivers, multidisciplinary health care providers, and health system leaders—will form an iKT panel. This iKT panel will collaborate with the research team to help inform patient facing study materials, recruitment and knowledge dissemination strategies of this research. This is particularly important as the perspectives of youth and caregiver knowledge users are paramount. These youth and caregiver knowledge users will be essential for developing plain language summaries of the research findings that are relevant and accessible to the public.

### Ethical Considerations

Ethics approval has been obtained from The Hospital of Sick Children (1000080241). The study has been reviewed and considered to be exempt by the RAND’s Human Subjects Protection Committee. Informed consent will be obtained before the start of the OMD study.

## Results

### Overview

The study is supported by funding from the CIHR through the Transitions in Care (TiC) Team Grant (funding reference number TRC 184533) and Physicians Services Incorporated (PSI; grant number 22-20). A 16-member iKT panel has been established to be actively involved in the preparation for the OMD. Enrollment will begin in September 2024 and the first round of the OMD will commence in October 2024. The study results are expected to be published in late 2026.

### Dissemination

We will leverage our partnerships with key knowledge users to promote rapid knowledge mobilization of the key quality indicators, with a primary focus on national-level knowledge dissemination to multiple relevant knowledge users. Our knowledge users have a proven track record of mobilizing knowledge to guide policy decisions and influence clinical practice. We will also engage primary care and community leaders through provincial- and national-level workshops, webinars, and media releases on the findings and implications for practice. Many members of the research team are affiliated or situated within pediatric and adult tertiary care and community clinics providing transition support and care to patients. As such, our results will be translated *directly* and *immediately* into these services.

Our study findings will be shared at national and international conferences and published in leading open access journals with youth and caregivers as coauthors. Together with our iKT panel, we will explore the information needs of our youth and families and ensure voices from diverse communities and populations are heard to realize a flexible and clear communication plan. Plain-language summaries with messages that are clear, simple, and individualized for each knowledge user group will be developed to augment the accessibility of the information.

## Discussion

### Anticipated Findings

Our innovative and equity-focused approach will reveal the differences and similarities in perceptions among youth, caregivers, health care providers, and health system leaders or decision makers on priority quality indicators for transition. The formation of an iKT panel that includes the most important knowledge users, the youth with chronic health conditions and caregivers, will allow the view of youth and caregivers to have significant weight in the study findings.

This study will define a candidate set of quality indicators for transition to adult care that are relevant, feasible to implement, and have the potential to improve the quality of care for Canadian youth and families. This work will respond to the lack of standardized quality indicators for transition from pediatric to adult care in Canada, which has been a major bottleneck toward achieving high quality care for young adults with chronic health conditions.

### Implications and Significance

This study will contribute readily applicable, adaptable, and relevant metrics to evaluate and benchmark transition outcomes nationally, addressing 3 major gaps in transition care and research. First, to reflect the needs and priorities of those receiving care, delivering services, and developing health care policies, we will engage with knowledge users involved in the continuum of transition care, including youth (ie, ages 18-24 years), caregivers, health care providers and health system leaders or decision makers. Using an iKT approach, we will appropriately engage and build collaborations with knowledge users throughout the research process [36]. Second, we will ensure demographic and sociocultural diversity among participants [45]. Nationally relevant benchmarks require representation from diverse geographical areas and practice settings [45]. To date, transition research has placed little emphasis on identifying the health system level practices that lead to inequitable transition care and subsequent negative health outcomes [47]. Our strong collaborations with key knowledge ensure that we are well positioned to recruit participants with diverse biological and sociocultural characteristics and lived experiences, including sex, gender, race or ethnicity, immigrant and refugee status, chronic disease, geography, and socioeconomic status [41], helping us to secure representation of diverse transition experiences from marginalized populations. Third, we will also focus on primary care integration. Adolescents with chronic health conditions should engage primary care services to meet their routine health care needs and maintain a continuous patient-provider relationship during the transition period to adult care [46]. An Ontario population–based cohort study by Toulany et al demonstrated youth with severe mental illness receiving no primary care during transition experienced an increased rate of mental health–related hospitalizations in young adulthood [48,49,51]. Despite this, transition research continues to focus on specialist providers [26,47].

### Strengths and Limitations

This research study has several notable strengths that contribute to the field of transition care. One strength of the OMD is to define a key of consensus-derived quality indicators that are not only meaningful to youth and families but also useful and applicable for use by health care providers and decision makers. Secondly, the iKT approach will foster collaborations with key knowledge users to ensure the needs and priorities of those receiving care, delivering services, and developing health care policies are reflected throughout the research and dissemination process. Finally, the online approach will allow for a large number of participants with wide geographical distribution and the anonymity of discussions will help promote equal participation without the influence of perceived hierarchy, ensuring that all voices are heard and valued.

This study is not without some potential limitations. While we recognize that internet accessibility may pose a barrier for some potential participants specifically in rural areas, choosing *ExpertLens* will allow greater participation across the country. This platform allows participants to connect to the platform using any basic internet-connected devices, including mobile phones and tablets, addressing some technological barriers that may arise. Using a mixed panel of different expert groups may pose a challenge. To address this issue, all study materials will be written in clear and concise language that is easily comprehensible for both youth and caregivers. Attrition is a potential limitation within a 3-round Delphi as participants may drop out after round 1. To encourage engagement in all 3 rounds, incentives will only be provided at the end.

### Conclusions

This study will produce a key set of quality indicators to evaluate and inform action to improve transition from pediatric to adult care for youth and their families equitably in Canada. Determining the most beneficial metrics from the perspectives of various knowledge users, most importantly youth and caregivers, is a fundamental step to identifying areas for improvement in transition, evaluating current processes, benchmarking across jurisdictions, and optimizing health outcomes for youth with chronic conditions into young adulthood.

## Appendix

Multimedia Appendix 1 Peer-review report.

## Abbreviations

ACCORD: ACcurate COnsensus Reporting Document
CHC: Children's Healthcare Canada
CIHR: Canadian Institutes of Health Research’s
CPS: Canadian Paediatric Society
iKT: Integrated Knowledge Translation
LGBTQQIP2SAA: lesbian, gay, bisexual, transgender, queer, questioning, intersex, pansexual, two-spirited, and asexual
OBI: Ontario Brain Institute
OMD: Online Modified Delphi
PCMCH: Provincial Council for Maternal and Child Health
PSI: Physicians Services Incorporated
RAM: RAND/UCLA Appropriateness Method
RPM: RAND/PPMD Patient-Centeredness Method
TiC: Transitions in Care

## Data Availability

The data sets generated and analyzed during this study are available from the corresponding author on reasonable request.
