# Supplementary material for: Quality Indicators for Transition from Pediatric to Adult Care for Youth With Chronic Conditions: Proposal for an Online Modified Delphi Study
Source: JMIR Res Protoc. 2024 Sep 10;13:e60860. doi: 10.2196/60860 (PMC11422730; doi:10.2196/60860)
Supplement: Multimedia Appendix 1 [file resprot_v13i1e60860_app1.pdf]

Review Type/Type d'évaluation: Committee Member 1/Membre de comité 1
Name of Applicant/Nom du chercheur: Toulany, Alene
Application No./Numéro de demande: 478918
Agency/Agence: CIHR/IRSC
Competition/Concours: 2022-01-25 Team Grant: Transitions in Care/Subvention d'équipe : Transitions dans les soins
Committee/Comité: Team Grant: Transitions in Care - - Phase 2/Subvention d'équipe : Transitions dans les soins - - Phase 2
Title/Titre: Quality Indicators for Transition from Paediatric to Adult Care for Youth with Chronic Physical, Developmental, and Mental Health Conditions: A National Consensus-Building Multi-stakeholder Initiative

---

Assessment/Évaluation:

Summary of the proposal

The overarching goal of this study is to establish a key set of consensus-derived quality indicators for transition to adult care that are applicable across chronic physical, developmental, and/or mental health conditions, clinical care settings, and health jurisdictions. These quality indicators are needed to evaluate health system performance and inform action to improve transition from paediatric to adult care for youth and their families equitably in Canada.

Specific Aims:

In Phase 1 of this study, the team aims to:

1. Establish a Project Advisory Committee (PAC) with diverse representation to refine an *initial* set of quality indicators previously identified for transition to adult care that will be prioritized by youth, parents /caregivers, health care providers, and health system and community leaders/decision makers.

In Phase 2 of this study, they aim to use an iterative, online modified-Delphi (OMD) process (120 experts: youth with physical, developmental, and/or mental health conditions; parents/caregivers; healthcare providers; and decision-makers) to:

2. Compare and contrast the perspectives of youth, parents/caregivers, health care providers, and health system and community leaders/decision-makers on quality indicators for transition that are most important, useful, and feasible.

3. Prioritize a set of quality indicators for transition applicable across chronic conditions and clinical care settings in the Canadian context.

Anticipated Outputs: This project will result in the formation of an iKT panel that includes the most important stakeholders, the youth with chronic health conditions and parents/caregivers. Placing significant weight on the views of youth and parents/caregivers, this study will define a candidate set of quality indicators for transition to adult care that are relevant, feasible to implement, and have the potential to improve the quality of care for Canadian youth and families. Their equity-focused approach will reveal the differences and similarities in perceptions among youth, parents/caregivers, healthcare providers, and health system and community leaders/decision-makers on priority quality indicators for transition.

Rational and Research Question

**Review Type/Type d'évaluation:**	Committee Member 1/Membre de comité 1
**Name of Applicant/Nom du chercheur:**	Toulany, Alene
**Application No./Numéro de demande:**	478918
**Agency/Agence:**	CIHR/IRSC
**Competition/Concours:**	2022-01-25 Team Grant: Transitions in Care/Subvention d'équipe : Transitions dans les soins
**Committee/Comité:**	Team Grant: Transitions in Care - - Phase 2/Subvention d'équipe : Transitions dans les soins - - Phase 2
**Title/Titre:**	Quality Indicators for Transition from Paediatric to Adult Care for Youth with Chronic Physical, Developmental, and Mental Health Conditions: A National Consensus-Building Multi-stakeholder Initiative

Assessment/Évaluation:

Strengths

- Transition from Paediatric to Adult Care for Youth with Chronic Physical, Developmental, and Mental Health Conditions is a priority.

- Proposing consensus-derived quality indicators is an important step to improve these transitions.

- The objectives respond to the objective of the funding opportunity.

- The team conducted a systematic review to inform their project (submitted for publication)

Weaknesses

-

<u>Planned intervention(s)/Solution(s) and Research Methodology</u>

Strengths

- The proposal is very clear.

- Methods are valid.

- The project addresses at least 2 focus areas of TiC.

- The focus on EDI is really present all over the proposal.

- The dissemination plan is strong.

Weaknesses

- Could have been useful to present a design with reference for phase 1 (participatory research for example).

- The proposal could have presented the challenges and mitigation strategies.

<u>Team</u>

**Review Type/Type d'évaluation:**	Committee Member 1/Membre de comité 1
**Name of Applicant/Nom du chercheur:**	Toulany, Alene
**Application No./Numéro de demande:**	478918
**Agency/Agence:**	CIHR/IRSC
**Competition/Concours:**	2022-01-25 Team Grant: Transitions in Care/Subvention d'équipe : Transitions dans les soins
**Committee/Comité:**	Team Grant: Transitions in Care - - Phase 2/Subvention d'équipe : Transitions dans les soins - - Phase 2
**Title/Titre:**	Quality Indicators for Transition from Paediatric to Adult Care for Youth with Chronic Physical, Developmental, and Mental Health Conditions: A National Consensus-Building Multi-stakeholder Initiative

Assessment/Évaluation:

Strengths

- Strategies to engage stakeholders are meaningful and well explained, and the governance structure is clear.

- They will apply the principles from the CIHR's Strategy for Patient-Oriented Research.

- The quality of the partnership is strong between the team and the partners.

- Applicants have the necessary qualifications to deliver planned outputs.

Weaknesses

-

Capacity Building and Research Environment

Strengths

- The proposal presents a clear capacity building plan.

- The research environment is well described and strong.

Weaknesses

-

Integration of Sex/Gender in the research proposal

Integration of Sex/gender as well as EDI principles is a strength of this proposal.

Review Type/Type d'évaluation: Committee Member 2/Membre de comité 2
Name of Applicant/Nom du chercheur: Toulany, Alene
Application No./Numéro de demande: 478918
Agency/Agence: CIHR/IRSC
Competition/Concours: 2022-01-25 Team Grant: Transitions in Care/Subvention d'équipe : Transitions dans les soins
Committee/Comité: Team Grant: Transitions in Care - - Phase 2/Subvention d'équipe : Transitions dans les soins - - Phase 2
Title/Titre: Quality Indicators for Transition from Paediatric to Adult Care for Youth with Chronic Physical, Developmental, and Mental Health Conditions: A National Consensus-Building Multi-stakeholder Initiative

---

Assessment/Évaluation:

Youths experience challenges in transition. An equity lens has not be incorporated into the development of quality indicators for youths in the past. Also, youths, especially those with mental health vulnerabilities have not been integrated into research on transition. This study aim to conduct a 3-round online modified-Delphi study to determine a key set of quality indicators for transition across chronic conditions that are most important, useful, and feasible from the perspective of youth and parents/caregivers with lived experiences, specialist and primary care clinicians, and health system and community leaders/decision-makers. Using an equity lens, the applicants will engage each stakeholder group to compare and contrast their perspectives on quality indicators for transition. The applicants will conduct a 3-round online modified-Delphi study to prioritize a set of quality indicators for transition across chronic physical, developmental, and mental health conditions that are most important, useful, and feasible for Canadians.

STRENGTH

Diverse stakeholders, including youths, will be engaged and contribute to all stages of the project

The project focuses on addressing equity. However, there needs to be more consideration of equity in analysis of results, who is trained and who is engaged on advisory committee

Project will collect data from youths.

WEAKNESS

Generally, I find this proposal is not robust and is overly prolonged and over budgeted.

No information given on the ability of youths with development and mental disabilities to complete and engage in this project.

Given a focus on addressing equity, the equity piece of this project including in knowledge mobilization, advisory committee selection, mentorship of early career researchers and data analysis need to be stronger. For instance, the projects central goal is to develop equity tools, however no language consideration is provided, including to immigrants and Francophone communities. Also, no information is provided on how the project will ensure youths from low income background who may not have access to internet or computer will be able to participate in this project.

This project is overly prolonged. All states of this project can easily be completed within 2 years given the

**Review Type/Type d'évaluation:**	Committee Member 2/Membre de comité 2
**Name of Applicant/Nom du chercheur:**	Toulany, Alene
**Application No./Numéro de demande:**	478918
**Agency/Agence:**	CIHR/IRSC
**Competition/Concours:**	2022-01-25 Team Grant: Transitions in Care/Subvention d'équipe : Transitions dans les soins
**Committee/Comité:**	Team Grant: Transitions in Care - - Phase 2/Subvention d'équipe : Transitions dans les soins - - Phase 2
**Title/Titre:**	Quality Indicators for Transition from Paediatric to Adult Care for Youth with Chronic Physical, Developmental, and Mental Health Conditions: A National Consensus-Building Multi-stakeholder Initiative

Assessment/Évaluation:

panel session will take less than a month to complete

A US online panel is being used. Information is not provided on how Canadian ethics approval will be ensured given storage of data in the US

Knowledge mobilization plan, including to equity seeking groups, need to be stronger given the focus on equity

BUDGET: This project is severely overbudgeted. The project can be completed for less than half of the budget amount. A research coordinator for four years at the current rate is not necessary for this project given the scope of the project. The coordinator salary can be cut by 150,000. Two years for a research assistant is sufficient. Research assistant cost can be cut by another 150,000. Expert Lens cost is too high and can be cut by half. Rather, funding should be allotted to areas in project that will help address equity gaps such as interpreter, translator and compensation or reimbursement to low income stakeholders.

**Review Type/Type d'évaluation:**	Committee Member 3/Membre de comité 3
**Name of Applicant/Nom du chercheur:**	Toulany, Alene
**Application No./Numéro de demande:**	478918
**Agency/Agence:**	CIHR/IRSC
**Competition/Concours:**	2022-01-25 Team Grant: Transitions in Care/Subvention d'équipe : Transitions dans les soins
**Committee/Comité:**	Team Grant: Transitions in Care - - Phase 2/Subvention d'équipe : Transitions dans les soins - - Phase 2
**Title/Titre:**	Quality Indicators for Transition from Paediatric to Adult Care for Youth with Chronic Physical, Developmental, and Mental Health Conditions: A National Consensus-Building Multi-stakeholder Initiative

Assessment/Évaluation:

Study aims to use Delphi methods to develop a series of quality indicators for transitions in care from paediatric to adult for chronic conditions. There is currently no consistent and universal way to measure quality across these transitions. The team plans to select these indicators with meaningful participation from youth and patients/caregivers with lived experience. First step in addressing a major challenge with transitions in care.

This proposal has a strong team with cross-country representation. Priority on balancing perspectives of clinicians and youth and parents/caregivers with lived experience is a strength of the proposal as well.

Existing relationships with knowledge users will help with knowledge translation and uptake of results of the study. Already a subcommittee of members with one of the knowledge users that is focused on quality indicators.

Uses well established methodology. No issues with the budget.

Review Type/Type d'évaluation: Committee Member 4/Membre de comité 4
Name of Applicant/Nom du chercheur: Toulany, Alene
Application No./Numéro de demande: 478918
Agency/Agence: CIHR/IRSC
Competition/Concours: 2022-01-25 Team Grant: Transitions in Care/Subvention d'équipe : Transitions dans les soins
Committee/Comité: Team Grant: Transitions in Care - - Phase 2/Subvention d'équipe : Transitions dans les soins - - Phase 2
Title/Titre: Quality Indicators for Transition from Paediatric to Adult Care for Youth with Chronic Physical, Developmental, and Mental Health Conditions: A National Consensus-Building Multi-stakeholder Initiative

---

Assessment/Évaluation:

<u>Competition:</u>CIHR Team Grant: Transitions In Care Phase 2 - 2022

<u>Project Application Title:</u> TG478918- Quality Indicators for Transition from Paediatric to Adult Care for Youth with Chronic Physical, Developmental, and Mental Health Conditions: A National Consensus-Building Multi-stakeholder Initiative

1. Research Question
a. Research question(s) is/are clearly stated. **- YES**

b. Strong scientific rationale for pursuing the proposed evaluation. **- YES**

c. Extent to which the research project responds to the objectives of the funding opportunity. **- STRONG**

2. Research Approach
a. Strength of the research approach and justification for the proposed methods/strategies that is supported by available evidence and/or literature. **– STRONG(See Comments)**

b. Appropriateness and rigor of the proposed study design to address the research question(s). **– STRONG (See Comments)**

c. Is the idea or approach novel, or is the work primarily confirmatory and/or a direct extension of previous work? **–See Comments**

d. Appropriate incorporation and justification of sex as a biological variable and/or gender as a social determinant of health where applicable. **– STRONG**
e. Appropriate incorporation of Indigenous culturally relevant theoretical and conceptual frameworks, and Indigenous culturally appropriate research protocols, including Indigenous methodologies where applicable. **– TBD By Assigned Experts**

**Review Type/Type d'évaluation:**	Committee Member 4/Membre de comité 4
**Name of Applicant/Nom du chercheur:**	Toulany, Alene
**Application No./Numéro de demande:**	478918
**Agency/Agence:**	CIHR/IRSC
**Competition/Concours:**	2022-01-25 Team Grant: Transitions in Care/Subvention d'équipe : Transitions dans les soins
**Committee/Comité:**	Team Grant: Transitions in Care - - Phase 2/Subvention d'équipe : Transitions dans les soins - - Phase 2
**Title/Titre:**	Quality Indicators for Transition from Paediatric to Adult Care for Youth with Chronic Physical, Developmental, and Mental Health Conditions: A National Consensus-Building Multi-stakeholder Initiative

Assessment/Évaluation:

3. Applicants
a. Strength of the applicants, taking into consideration evidence that there is the appropriate expertise, influence, resources and stakeholders who are appropriately and meaningfully involved. **–STRONG (See Comments)**

b. Appropriate engagement of the knowledge user(s) responsible for, or involved in decision-making of, the activity being evaluated. **–MODERATE(See Comments)**

4. Feasibility
a. Appropriateness of the budget and the justification for the amount requested, including the required budget for compensation of meaningfully engaged patient partnerships, as well as to cover knowledge translation/dissemination activities. **– MODERATE(See Comments)**

b. Suitability of the environment, including availability and accessibility of personnel and tools, to conduct the proposed activities. **– STRONG (See Comments)**

c. Probability that the project objectives will be met within the proposed timeline. - **STRONG**

5. Impact of Research
a. Strength of the dissemination plan. **– STRONG (See Comments)**

b. Potential of the proposal to advance knowledge and produce high-quality evidence to inform actionable health system changes to improve care transitions. **– STRONG (See Comments)**

<u>Notes/Comments For Applicants:</u>

-Promising overall methods, knowledge translation/implementation and evaluation plan described.

-Sex/gender and multiple metrics of demographic diversity are inclusively embedded.

-Allocated budget will help remove some identified potential barriers to participation (e.g.

Review Type/Type d'évaluation: Committee Member 4/Membre de comité 4
Name of Applicant/Nom du chercheur: Toulany, Alene
Application No./Numéro de demande: 478918
Agency/Agence: CIHR/IRSC
Competition/Concours: 2022-01-25 Team Grant: Transitions in Care/Subvention d'équipe : Transitions dans les soins
Committee/Comité: Team Grant: Transitions in Care - - Phase 2/Subvention d'équipe : Transitions dans les soins - - Phase 2
Title/Titre: Quality Indicators for Transition from Paediatric to Adult Care for Youth with Chronic Physical, Developmental, and Mental Health Conditions: A National Consensus-Building Multi-stakeholder Initiative

---

Assessment/Évaluation:

transportation, etc.)
-Patient/Parent Partner compensation plan articulated in the budget may need to be revised in the event of expanded time-commitment/workload.

-No circle of health system and/or community support letters were enclosed with this application.

-No youth/parent partner skills/experience backgrounders, nor attestations describing the scope of each individual's planned contributions to engagement activities, nor expressing personal support for the (cohesive and appropriately resourced?) conduct of the research were enclosed with this application.

Review Type/Type d'évaluation: SO Notes /Notes de l'agent scientifique
Name of Applicant/Nom du chercheur: Toulany, Alene
Application No./Numéro de demande: 478918
Agency/Agence: CIHR/IRSC
Competition/Concours: 2022-01-25 Team Grant: Transitions in Care/Subvention d'équipe : Transitions dans les soins
Committee/Comité: Team Grant: Transitions in Care - - Phase 2/Subvention d'équipe : Transitions dans les soins - - Phase 2
Title/Titre: Quality Indicators for Transition from Paediatric to Adult Care for Youth with Chronic Physical, Developmental, and Mental Health Conditions: A National Consensus-Building Multi-stakeholder Initiative

---

Assessment/Évaluation:

Rationale and Research Question : The proposal attempts to develop quality care indicators across the transition from pediatric to adult care. The importance of this work was noted as a strength. The team has completed a previous systematic review which further underscores the gap to be filled.

Planned Intervention(s)/Solution(s) and Research Methodology : The incorporation of equity, diversity and inclusion was discussed. It was noted that the focus of the project was EDI and some there are missing elements. The proposal integrated EDI in the research but it is not grounded through an EDI approach to research. For example, the knowledge mobilization plan did not outline how this work will be translated to the youth community which was a notable gap. The proposal would benefit from consideration of language barriers and income disparities.

More details and clarity on how will the youth and parent partners will be included were required. There was also discussion about how engaged the youth and parents are given that there are no support letters within the application.

Another missing piece noted was consideration about how the ethical considerations of gathering and storing data from the US panel will be addressed. The timeline for the project was discussed with some noting the timeline could be shortened. More clarity about the timelines and milestones of the project would support the 4 year timeline proposed.

Team : The team is excellent.

Capacity Building and Research Environment : The capacity building plan is viewed as a strength with clear details and a well-articulated plan.

Sex and Gender: Well integrated and considered.

The inclusion of transportation costs was important. The patient partner compensation may need to be reconsidered, and increased given the possibility of the time commitment expanding. The administrative human resources were viewed as a high. Specifically, the inclusion of a full time and part time research coordinator for the entire duration of the project was felt to be over-resourced. Similarly the number of research assistants could reduced and the expert lens costs were felt to be too high. One of the missing pieces was a project manager/liaison with the participants in the project. This was felt to be an important role to interface and support the participant's ability to contribute to the work. The committee recommended reallocating one of the research coordinators to a role that supported the participants and focused on the EDI aspects and the reciprocity required for this extensive community engagement.

****

Note: The final rating of the application, provided in the Notice of Decision (NOD), is the averaged rating of the peer review committee members following the discussion of the application during the committee meeting, and therefore may differ from the ratings provided by the assigned reviewers in their respective reviews.

**Review Type/Type d'évaluation:**	SO Notes /Notes de l'agent scientifique
**Name of Applicant/Nom du chercheur:**	Toulany, Alene
**Application No./Numéro de demande:**	478918
**Agency/Agence:**	CIHR/IRSC
**Competition/Concours:**	2022-01-25 Team Grant: Transitions in Care/Subvention d'équipe : Transitions dans les soins
**Committee/Comité:**	Team Grant: Transitions in Care - - Phase 2/Subvention d'équipe : Transitions dans les soins - - Phase 2
**Title/Titre:**	Quality Indicators for Transition from Paediatric to Adult Care for Youth with Chronic Physical, Developmental, and Mental Health Conditions: A National Consensus-Building Multi-stakeholder Initiative

Assessment/Évaluation:

Remarque : La cote définitive de la demande, qui apparaît dans l’avis de décision, représente la moyenne des cotes accordées par les membres du comité d’ évaluation par les pairs après avoir débattu de la demande à la réunion du comité. Elle peut donc différer de celle donnée par les évaluateurs dans leur évaluation respective.

````````````````````````````````````````````````````````````````````````````````````````````````````````````````````````````````````````````````````````````````````
````````````````````````````````````````````````````````````````````````````````````````````````````````````````````````````````````````````````````````````````````
